# Understanding Ameliorating Effects of Boron on Adaptation to Salt Stress in Arabidopsis

**DOI:** 10.3390/plants13141960

**Published:** 2024-07-17

**Authors:** Mei Qu, Xin Huang, Lana Shabala, Anja Thoe Fuglsang, Min Yu, Sergey Shabala

**Affiliations:** 1 International Research Center for Environmental Membrane Biology, Foshan University, Foshan 528000, China; mei.qu@utas.edu.au (M.Q.); xin.huang@fosu.edu.cn (X.H.); l.shabala@utas.edu.au (L.S.); 2 Tasmanian Institute of Agriculture, University of Tasmania, Hobart 7005, Australia; 3 Department of Plant and Environmental Sciences, University of Copenhagen, 1871 Copenhagen, Denmark; atf@plen.ku.dk; 4 School of Biological Sciences, University of Western Australia, Perth 6009, Australia

**Keywords:** NaCl, boron, Arabidopsis, H^+^, K^+^, halotropism

## Abstract

When faced with salinity stress, plants typically exhibit a slowdown in their growth patterns. Boron (B) is an essential micronutrient for plants that are known to play a critical role in controlling cell wall properties. In this study, we used the model plant *Arabidopsis thaliana* Col-0 and relevant mutants to explore how the difference in B availability may modulate plant responses to salt stress. There was a visible root growth suppression of Col-0 with the increased salt levels in the absence of B while this growth reduction was remarkably alleviated by B supply. Pharmacological experiments revealed that orthovanadate (a known blocker of H^+^-ATPase) inhibited root growth at no B condition, but had no effect in the presence of 30 μM B. Salinity stress resulted in a massive K^+^ loss from mature zones of *A. thaliana* roots; this efflux was attenuated in the presence of B. Supplemental B also increased the magnitude of net H^+^ pumping by plant roots. Boron availability was also essential for root halotropism. Interestingly, the *aha2Δ57* mutant with active H^+^-ATPase protein exhibited the same halotropism response as Col-0 while the *aha2-4* mutant had a stronger halotropism response (larger bending angle) compared with that of Col-0. Overall, the ameliorative effect of B on the *A. thaliana* growth under salt stress is based on the H^+^-ATPase stimulation and a subsequent K^+^ retention, involving auxin- and ROS-pathways.

## 1. Introduction

More than 1125 million hectares of land worldwide are affected by salt [1]. This amount represents over 7% of the total land area in the world. Soils are categorized as saline when electric conductivity (ECe) is 4 dS/m or higher [2]. Soil salinization causes osmotic stress, ion toxicity, and oxidative stress which threatens crop productivity, and salinity is particularly prevalent in arid and semi-arid regions of the world due to insufficient rainfall and inadequate water and soil management practices [3,4]. Salinity stress is mostly associated with NaCl–a salt with the highest solubility in water. Its presence in the soil causes osmotic stress and reduces cell turgor (hence, expansion growth), and excessive accumulation of Na^+^ and Cl^−^ in plant tissues interferes with cell metabolism [5,6]. Increased Na^+^ absorption induces plasma membrane depolarization causing K^+^ efflux under saline conditions, and salinity-induced programmed cell death (PCD) in plant roots is causally linked to cytosolic K^+^ depletion [7]. In addition, elevated salinity levels impede the electron flow from the central transport chain to the oxygen reduction pathways within various organelles, resulting in an excessive generation of reactive oxygen species (ROS) in plants [8], which has detrimental effects on cell expansion [9], photosynthesis [10,11], and ion (Na^+^, K^+^, and Ca^2+^) homeostasis [12,13]. Enhancing the ability of plants to tolerate high levels of salt is of great significance in promoting sustainable agriculture. The capacity of plants to adapt or tolerate salinity stress requires the involvement of intricate physiological characteristics, metabolic pathways, and molecular or genetic networks, which should be correctly coordinated. The alternative option is to mitigate salinity stress via agronomy means. One of them may be to optimize plant nutrition.

Boron (B) is a chemical element with the atomic number 5 and an atomic weight of 10.821. As the essential nutrient, B functions in many aspects, including maintaining cell wall and membrane integrity, promoting cell division and elongation, facilitating reproductive growth, synthesizing carbohydrates and proteins, metabolizing phenols and auxins, managing disease resistance, and adapting to various abiotic stress [14]. Boric acid exists in a neutral, uncharged form (H_3_BO_3_) at physiological pH. Its uptake by plant roots is primarily a passive process, which is largely influenced by the rate of water absorbed by the root cells [15] and occurs through specialized water channels (aquaporins) such as NIP (nodulin 26 (NOD26)-like intrinsic proteins) [16]. Plants also possess specialized active boron transporters (BOR1; [17]). The requirement for B varies greatly between plant species, and the range between B deficiency and B toxicity is relatively narrow. Hence, it is crucial to control optimal B levels in the soil which is significant for enhancing crop production.

The positive impact of B supply on alleviating NaCl stress can be attributed to various factors. When subjected to salt stress, exogenous application of appropriate B resulted in better plant growth of wheat [18], maize [19], broccoli [20], and cotton [21], which was attributed to the increase of K content and a reduction in Na and Cl content. B supply significantly alleviated the detrimental effects of salt stress in plants by enhancing relative water content and soluble carbohydrate levels in the leaves which effectively maintained the crucial osmotic potential required for optimal plant functioning [22]. Besides, the adequate B supply alleviated the inhibition of photosynthesis caused by salinity through the increase of chlorophyll and total chlorophyll content in purslane (*Portulaca oleracea* L.) [23], and this amelioration of B on photosynthesis under saline conditions also has been reported in sunflower [24]. The presence of B enhanced the activities of antioxidant defense systems, which ameliorated the salt-induced oxidative stress and improved the physiological parameters of soybean plants [25,26].

However, most of the reported studies have been essentially observational and failed to reveal the mechanistic basis for B control of key adaptive traits allowing plants to adapt to saline conditions. Our previous work showed that B ameliorated detrimental salinity effects in barley and attributed it to B control of H^+^-ATPase, membrane potential maintenance, and Na^+^ exclusion [27]. However, that work was done on barley–one of the few most tolerant crop species. Can these findings be extrapolated to more salt-sensitive species? And what was the genetic basis of the above processes? To answer these questions, we combined some complementary approaches (physiological, biophysical, and genetic) to study the ameliorative effects of B on the performance of *Arabidopsis thaliana*; a species that is highly sensitive to salt stress [28] and that is used as a model organism due to availability of a large number of various mutants. The obtained results suggest that the ameliorative effect of B on the *A. thaliana* growth under salt stress is based on the H^+^-ATPase stimulation and a subsequent K^+^ retention, via auxin- and ROS-mediated pathways.

## 2. Results

### 2.1. Effect of Salt on the Growth of A. thaliana

**Figure 1:**
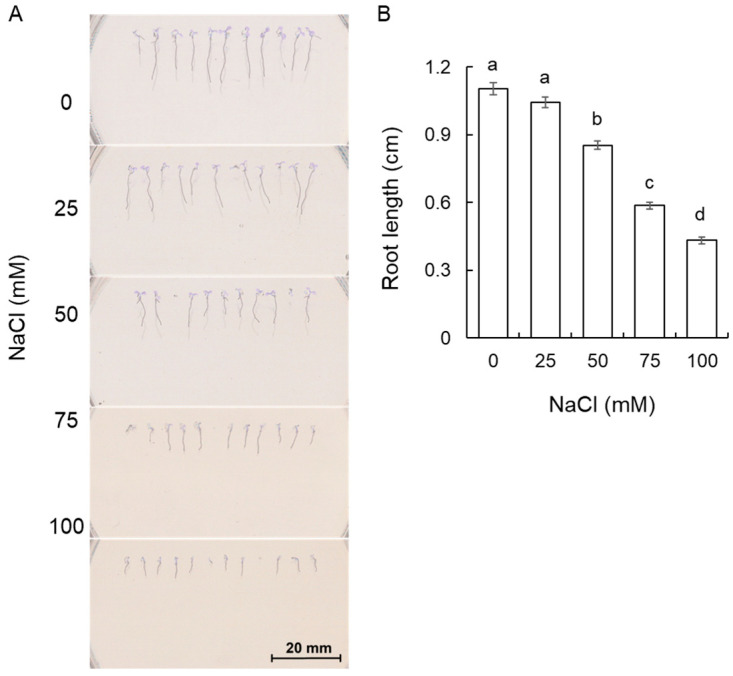
Effect of NaCl on phenotype (**A**) and root length (**B**) of *A. thaliana* Col-0 plants. Data are mean ± SE (n = 8–12). One-way ANOVA followed by Tukey’s post-hoc test was conducted for statistical analysis. Data labeled with different low-case letters are significantly different at *p* < 0.05.

Salt stress can affect a myriad of plants’ morphological, biochemical, and physiological processes, leading to a decrease in biomass production. In the beginning, the different salt concentrations were screened to observe the degree of inhibition of the root growth. In the absence of B, as the concentration of salt increased, *A. thaliana* Col-0 root growth declined (Figure 1A), and 100 mM NaCl resulted in 60.8% root length suppression, while 25 mM NaCl showed no significant effect on root growth (Figure 1B). Accordingly, 100 mM NaCl was selected for future experiments.

### 2.2. The Role of Boron in the Growth of A. thaliana in the Presence of Salt

**Figure 2:**
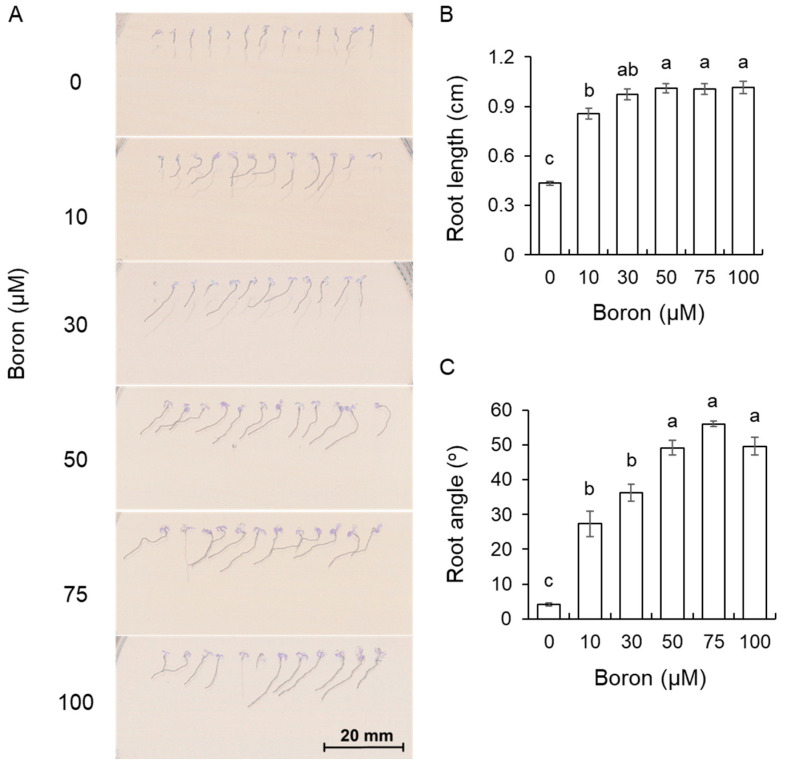
Effect of boron on phenotype (**A**), root length (**B**), and root angle (**C**) of *A. thaliana* Col-0 plants grown in the presence of 100 mM NaCl. Data are mean ± SE (n = 8–12). One-way ANOVA followed by Tukey’s post-hoc test was conducted for statistical analysis. Data labeled with different low-case letters are significantly different at *p* < 0.05.

Effects of salinity on root growth were most detrimental in the absence of B. When B was added to the growth media (10–100 μM range), plant performance was significantly improved (Figure 2A), and 30 μM B resulted in twice longer roots compared with no B treatment (Figure 2B). Interestingly, the presence of B roots exhibited some clear signs of halotropism, deviating by 35° to 55° from the vertical growth in saline media (Figure 2C). The effect was dose-dependent, with the highest deviation reported for 75 μM B treatment.

### 2.3. Potential Signaling Pathway Involved in Ameliorating Effects of Boron

**Figure 3:**
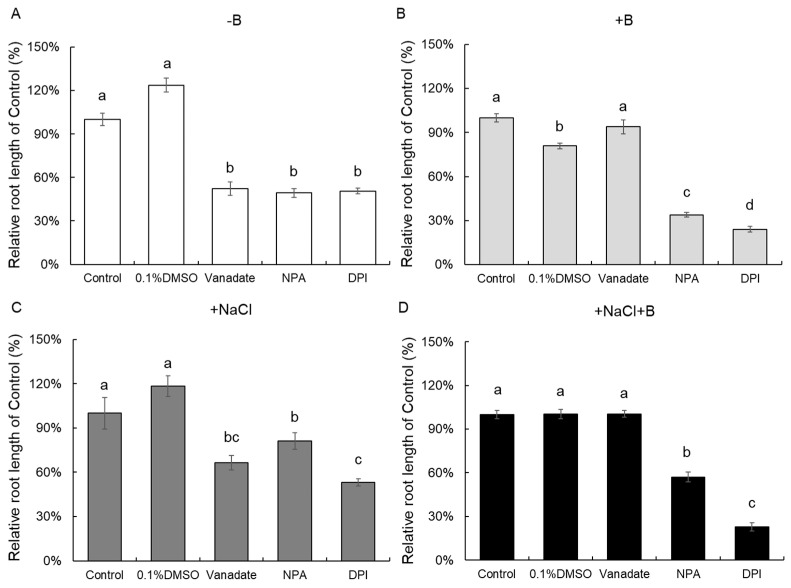
Effect of inhibitors on the relative root length of *A. thaliana* Col-0 plants in the presence of different boron and salt treatments. Data are mean ± SE (n = 8–12). One-way ANOVA followed by Tukey’s post-hoc test was conducted for statistical analysis. Data labeled with different low-case letters are significantly different at *p* < 0.05.

To understand the possible downstream targets of B, we used inhibitors of different signaling pathways and effectors (transporters) to investigate the mechanistic basis of alleviating the effects of B on root growth under salt stress (Figure 2). Vanadate is a known inhibitor of ATPases and other ATP hydrolyzing enzymes, and we observed the reduced growth of roots was recovered in the presence of 30 μM B regardless of 100 mM NaCl (Figure 3). The growth of roots was also reduced in the presence of N-1-naphthylphthalamic acid (NPA; an inhibitor of the polar auxin transport) and DPI (a blocker of NADPH oxidase) although the presence of B did not alleviate this inhibition (Figure 3).

### 2.4. Regulation of H^+^-ATPase Associated with Halotropism

**Figure 4:**
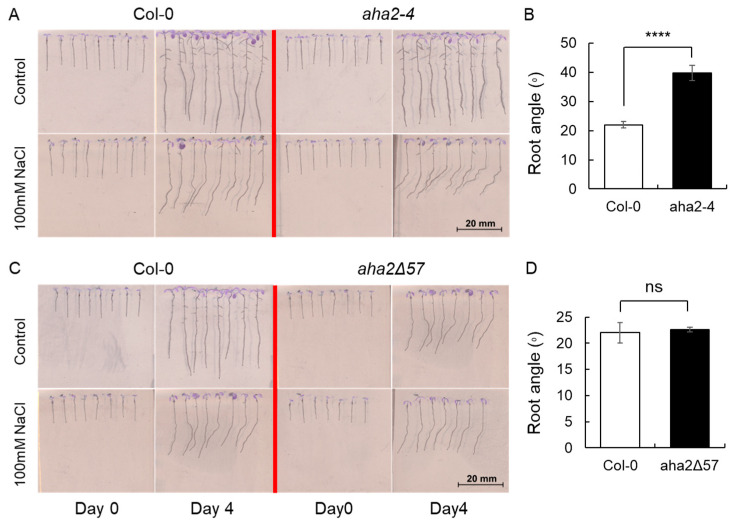
Effect of salt on phenotype (**A**,**C**) and root angle (**B**,**D**) of *A. thaliana* plants. Data are mean ± SE (n = 8–12). A *t*-test was conducted for statistical analysis. Data is significant at **** *p* < 0.0001, and ns = not significant at *p* < 0.05.

Halotropism is a response of plant roots to avoid a saline environment. In the presence of B, roots of *A. thaliana* Col-0 showed a bending growth pattern when grown in the presence of 100 mM NaCl (Figure 4A). Cell elongation requires an active plasma membrane (PM) H^+^-ATPase, so we investigated this by employing a deletion mutant *aha2-4* and a mutant encoding a constitutively active version, *aha2∆57*, lacking part of the regulatory domain. The *aha2-4* deletion mutant exhibited an even stronger halotropism than that of the wild-type (the bending angle of 40° vs. 22°, respectively (Figure 4B). At the same time, the *aha2Δ57* mutant exhibited the same halotropic patterns as the wild type (Figure 4D).

### 2.5. Dose Dependence of Boron H^+^ and K^+^ Response to NaCl

**Figure 5:**
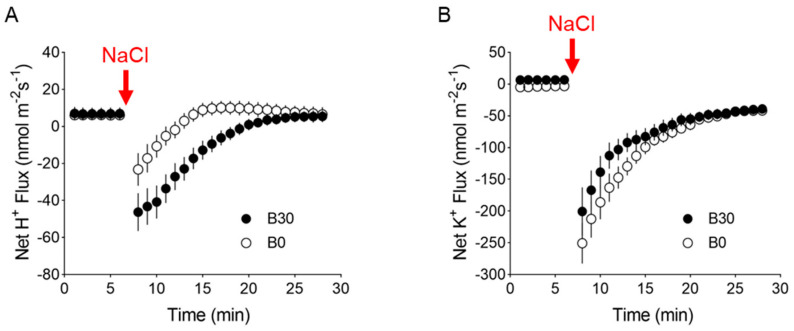
Transient net H^+^ (**A**) and K^+^ (**B**) flux measured mature root zone of *A. thaliana* Col-0 plants in response to acute 100 mM NaCl treatment. Mean ± SE (n = 6–8). The sign convention is efflux negative.

We then conducted electrophysiological experiments to further explore the role of H^+^-ATPase in B-mediated mitigation of salt stress. Plants pre-treated with 30 μM B for 3 h showed higher H^+^ efflux (a proxy for H^+^-ATPase activity) upon transient 100 mM NaCl addition as compared with-B plants (Figure 5A), indicating the stimulation of H^+^-ATPase by B. Also, smaller NaCl-induced K^+^ efflux demonstrated that B pretreatment was essential for improved potassium retention capacity (a key component of the salinity tissue tolerance mechanism) (Figure 5B).

## 3. Discussion

The initial perception of salt stress occurs in the roots and results in an immediate root growth inhibition, primarily due to the osmotic stress resulting from limited water availability. Days and weeks later, ion toxicity caused by an imbalance of nutrients in the cytosol due to excessive uptake of Cl^−^ and Na^+^ starts to prevail [29,30]. Under extended periods of salt stress, plants produce reactive oxygen species (ROS) in both roots and shoots, and it is commonly linked to the effects of salinity [31], with a series of adverse effects occurring successively, ultimately affecting the normal growth and development of plants. *A. thaliana* has been found to have a moderate level of salt tolerance [28], and the growth of the *A. thaliana* primary root is inhibited by salt stress, which a decrease in cell production and a smaller mature cell length are linked to the process of this reduction [32].

Boron functions in many aspects, including maintaining cell wall and membrane integrity, promoting cell division and elongation, facilitating reproductive growth, synthesizing carbohydrates and proteins, metabolizing phenols and auxins, managing disease resistance, and adapting to various abiotic stress [14]. Boron promotes plant growth in different plant species which requires an appropriate amount [33,34]. Exogenous B leads to a decrease in Cl^−^ content in sugar beet seedlings, thus improving plant performance in saline soils [35]. The ameliorating effect of boron on salt stress was also observed in barley plants, as reflected by better growth with improved photosynthesis [27]. However, most of the reported studies have been essentially observational and failed to reveal the mechanistic basis for B control of key adaptive traits allowing plants to adapt to saline conditions.

After being activated, the PM H^+^-ATPase functions to acidify the apoplast, subsequently triggering the activation of enzymes responsible for cell wall loosening thus facilitating the cell elongation [36]. Orthovanadate inhibited root growth at no B condition, while there was no inhibitory effect with 30 μM B addition (Figure 3), suggesting a causal link between H^+^-ATPase operation, boron availability, and expansion growth. B deficiency resulted in the suppression of the vanadate-sensitive H^+^-ATPase activity and H^+^ efflux in sunflower root microsomes, as opposed to the normal B condition [37]. The transcript levels of tobacco root plasma membrane H^+^-ATPase 2 (PMA2) increased with B supply, potentially resulting in an elevation of the PM H^+^-ATPase activity required for pumping protons out of the cell [38], and the higher H^+^-ATPase activity is required to provide the H^+^ gradient for activating Na^+^/H^+^ antiporter excluding excessive Na^+^ to improve salt tolerance [39].

Halotropism enables plant seedlings to minimize their exposure to salinity by avoiding direct contact with the saline environment [40]. Typically, the presence of high salt levels leads to the activation of phospholipase Ds (PLDs) PLDζ2, the internalization and recycling of PIN-FORMED 2 (PIN2) auxin efflux carrier [40], meanwhile, the changes in AUX1 auxin influx carrier polarity leading to an uneven distribution of auxin thus the root bend away from the salt [41,42]. The PM structure is stabilized by B through the formation of complexes with its constituents (glycolipids or glycoproteins), leading to the stabilization of enzymes or channels in an optimal conformation while being securely anchored to the membrane [43]. Halotropism was more pronounced in plants lacking functions H^+^-ATPase (*aha2-4* mutant) but not different in *aha2Δ57* mutant with active pump protein. It was shown that *A. thaliana* DNAJ HOMOLOG 3 (J3) mutants exhibit decreased PM H^+^-ATPase activity and H^+^ efflux and are hypersensitive to salinity [44]. The chaperone protein J3 enhances PM H^+^-ATPase activity by repressing SOS2-LIKE PROTEIN KINASE5 (PKS5), which phosphorylates the *A. thaliana* PM H^+^-ATPase AHA2 and prevents the binding of 14-3-3 proteins to AHA2, leading to an inactivation of H^+^-ATPase activity. The auxin exporter PIN2 participates in PKS5-mediated alkaline-stress responses by regulating PM H^+^-ATPase activity and proton fluxes from root apices. The primary roots of *A. thaliana pin2* and *pin2/pks5* mutants both secrete fewer protons and are hypersensitive to alkaline stress [45]. Exposure to salinity has been demonstrated to trigger the internalization (endocytosis) of PIN2 at the side of the root facing the higher salt concentration. Since the gravitropic response is controlled by PIN2-mediated redistribution of auxin that leads to the asymmetry of H^+^ fluxes and cell elongation between the upper and the lower side of the roots [46,47], it is reasonable to anticipate that H^+^ fluxes may also be crucial in regulating root halotropism. Boron supply promotes PIN2 endosome-based auxin transport to alleviate Al toxicity in plant roots [48], and boron also stimulates PM H^+^-ATPase with the ATP hydrolysis and H^+^ transport activity increased of lily pollen grains [49]. Therefore, further studies need to be done to explore the link of boron, auxin, and H^+^-ATPase mediating halotropism.

The survival of plants under saline conditions largely relies on their capacity to maintain ionic homeostasis. Under salt stress, plant cells need to employ primary active transport, mediated by H^+^-ATPases, and secondary transport, mediated by channels and co-transporters, to maintain characteristically high concentrations of K^+^ and low concentrations of Na^+^ in the cytosol, which is important for the activities of many cytosolic enzymes in plant cells [50]. During salt stress, K^+^ loss through depolarization-activated K^+^ outward-rectifying (KOR) channels and non-selective cation channels (NSCCs) resulted in the decline of membrane potential [51]. The improved plant salinity tolerance attributed to better K^+^ retention was also reported in wheat [52], quinoa [53], and *Cucurbita* species [54]. The observed hyperpolarization of root cell membranes in sunflowers may be a result of the stimulation of the H^+^-ATPase attributed to the increase in K^+^ uptake induced by the B supply [55]. The more negative membrane potential attributed to B supply leads to more H^+^ efflux and less K^+^ efflux in the barley root which confirms the ameliorating effect of B under salinity [27]. This was also the case here with *A. thaliana* (Figure 5). Thus, the ability of B to stimulate H^+^-ATPase activity seems to be an essential component of the tissue tolerance mechanism, via restoring membrane potential and improved K^+^ retention [56].

B-mediated control of H^+^-ATPase is also essential for root halotropism. Several factors were reported to be involved in the regulation of halotropism including light [57], ABA [58], and salt-specific genetic components (transcription factor WRKY25, cation-proton exchanger CHX13) [59]. Their causal links a separate investigation.

**Figure 6:**
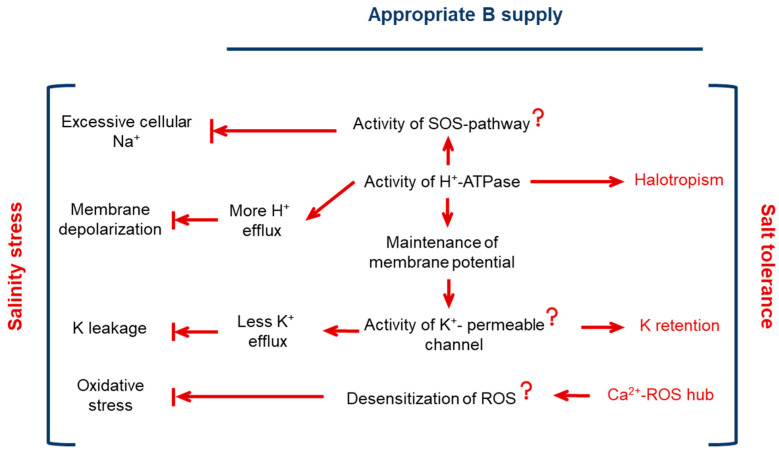
Suggested model of B-mediated amelioration of salinity stress tolerance. Central to this process is B-mediated activation of H^+^-ATPase with the consequences for (i) cytosolic K^+^ retention; (ii) Na^+^ exclusion from metabolically active compartments; and (iii) stress avoidance via halotrppism. The causal role of B in regulating plant redox balance (oxidative stress component) was not discussed in detail in this work but explicitly addressed in our previous studies [60]. It is also evident from the reported negative effects of DPI (a known inhibitor of NADPH oxidase) on root growth shown in Figure 3.

The overall summary of B-mediated regulation of H^+^-ATPase and its implication for plant ionic homeostasis and root growth under saline conditions is given in Figure 6.

## 4. Materials and Methods

### 4.1. Plant Material, Growth Conditions, and Treatment

Seeds of *A. thaliana* wild type (WT) Columbia-0 (Col-0), *A. thaliana* loss-of-function mutant *aha2-4* were obtained from the Arabidopsis Biological Resource Centre (http://www.Arabidopsis.org/abrc/, accessed on 18 June 2024), and *aha2Δ57* mutant with 57 residues being truncated at regulatory c-term by CRISPR, which was obtained from our lab [61]. The seeds were surface sterilized with 1 mL of commercial bleach (1% *v*/*v* NaClO) for 10 min and then washed at least five times with sterilized distilled water. Seeds were kept at 4 °C for 2 days and sown in Petri dishes containing modified half-strength Murashige and Skoog (MS) with 0.8% (*w*/*v*) Gellan Gum and 1% (*w*/*v*) sucrose at pH 5.7. Petri dishes containing seeds were sealed with parafilm and then transferred into a growth chamber (Fitotron Pro-Face, Canada, Greensboro, NC, USA) with 16 h/8 h day/night length, 100 µmol m^−2^ s^−1^ photon flux density, at 22 °C. The Petri dishes were oriented upright allowing the roots to grow down along the surface without penetrating the medium. All chemicals were from Sigma-Aldrich (Castle Hill, NSW, Australia) in analytical grade unless individually specified. The water we used to prepare the required solution was boron removed by borate-specific chelating resin (Amberlite^®^ IRA743 free base, Sigma-Alcrich, St. Louis, MI, USA).

For the salt screening experiment, sterilized seeds were directly sown in Petri dishes containing modified half-strength (MS) and different salt concentrations (NaCl, 0–100 mM) with 0.8% (*w*/*v*) Gellan Gum and 1% (*w*/*v*) sucrose at pH 5.7. For the boron (B) screening experiment, sterilized seeds were directly sown in Petri dishes containing modified half-strength (MS) and different B concentrations (H_3_BO_3_, 0–100 μM) plus 100 mM NaCl with 0.8% (*w*/*v*) Gellan Gum and 1% (*w*/*v*) sucrose at pH 5.7. For halotropism studies, seeds were sown in Petri dishes containing modified half-strength (MS) with 0.8% (*w*/*v*) Gellan Gum and 1% (*w*/*v*) sucrose at pH 5.7 for 3–4 days of normal growth before transferring to Petri dishes with different salt (NaCl, 0/100 mM) treatments. The pharmacological-related experiment was conducted by sowing seeds directly in Petri dishes containing modified half-strength (MS) and different B concentrations (H_3_BO_3_, 0/30 μM) and salt (NaCl, 0/100 mM) plus inhibitors (5 μM Vanadate, 3 μM N-1-naphthylphthalamic acid (NPA), 0.3 μM Diphenyleneiodonium chloride (DPI) separately) and 0.1% DMSO with 0.8% (*w*/*v*) Gellan Gum and 1% (*w*/*v*) sucrose at pH 5.7.

### 4.2. Plant Growth Parameters

The phenotype was recorded by the scanner (Epson Perfection V550 Photo, Tokyo, Japan), and the root length and root angle were measured directly or before and after transferring to new Petri dishes by Image J1 software.

### 4.3. Ion Flux Measurements

Net fluxes of H^+^ and K^+^ were measured from the root mature zone by using the non-invasive Microelectrode Ion Flux Estimation (MIFE) technique, with both the principle and procedure described in previous publications [62,63,64]. Six-day-old plants were immobilized in the Perspex measuring chamber containing fresh BSM (0.5 mM KCl and 0.1 mM CaCl_2_) solution to allow acclimating for 60 min. Steady-state net ion fluxes were then measured for 5–10 min, before adding 100 mM NaCl followed by another 30 min recording. For data analysis, the following parameters were calculated: ∆peak flux: a difference between the initial (steady state) flux value and a peak value in response to the transient NaCl application.

### 4.4. Statistical Analysis

The statistical analysis was analyzed by SPSS software (version 26, Chicago, IL, USA) and GraphPad Prism software (version 9.0, Dotmatics, Boston, MA, USA). All significant comparison was determined by one-way ANOVA followed by Tukey’s post-hoc test was conducted for statistical analysis except Figure 4 which was determined by a *t*-test.

## 5. Conclusions

In *A. thaliana*, the beneficial effect of B on the growth under salt stress is causally related to its control of H^+^-ATPase activity, with implications for membrane potential maintenance and, hence, cytosolic K^+^ retention (tissue tolerance). B control of H^+^-ATPase activity is also essential for stress avoidance through halotropism that is causally linked with B-controlled redistribution of auxin. The ability of plant roots for apoplastic ROS production by NADPH oxidase also appears to be essential for its adaptation to a saline environment.

## Data Availability

Data is contained within the article.
